# Ethanol Blocks the Reversal of Prolonged Dopamine Inhibition of Dopaminergic Neurons of the Ventral Tegmental Area

**DOI:** 10.1111/j.1530-0277.2012.01814.x

**Published:** 2012-05-02

**Authors:** Sudarat Nimitvilai, Devinder S Arora, Maureen A McElvain, Mark S Brodie

**Affiliations:** Department of Physiology and Biophysics, University of Illinois at ChicagoChicago, Illinois

**Keywords:** D2 receptor, Desensitization, Protein Kinase C, Electrophysiology, Brain Slices

## Abstract

**Background:**

Dopaminergic (DAergic) neurons of the ventral tegmental area (VTA) are important for the rewarding and reinforcing properties of alcohol and other drugs of abuse. Regulation of the firing of DAergic VTA neurons is controlled by a number of factors, including autoregulation of firing by D2 dopamine (DA) receptors. The inhibitory effects of DA on these neurons exhibit concentration- and time-dependent desensitization, which we have termed dopamine inhibition reversal (DIR), as it requires concurrent stimulation of D1/D5 and D2 receptors.

**Methods:**

Extracellular recording of DAergic VTA neurons in brain slices was used to test the effects of ethanol (EtOH) (10 to 80 mM) on DIR.

**Results:**

DIR was reduced by concentrations of EtOH as low as 10 mM and was blocked by higher EtOH concentrations. In addition, as we have shown that reversal of inhibition by the selective D2 agonist quinpirole can be observed in the presence of an activator of protein kinase C (PKC), we tested whether EtOH could antagonize the reversal of quinpirole inhibition in the presence of phorbol 12-myristate 13-acetate (PMA). EtOH (80 mM) blocked the reversal of quinpirole seen in the presence of PMA, suggesting that the antagonism of DIR by EtOH is owing to an action at a stage in the mechanism at or distal to PKC. Once achieved, DIR is not antagonized by EtOH.

**Conclusions:**

The blockade by relatively low concentrations of EtOH of DIR may play an important role in the spectrum of action of EtOH on DAergic neurons of the VTA and may be important in the acute and chronic actions of EtOH on the excitability of these brain reward/reinforcement neurons.

Neurons of the ventral tegmental area (VTA) project to several regions of the extended amygdala including the nucleus accumbens, prefrontal cortex, and amygdala (Koob, 2003). Dopaminergic (DAergic) projections from neurons of the VTA participate in the mediation of the rewarding/reinforcing properties of numerous drugs of abuse (Wise, 1996). The dopamine (DA) neurons themselves are regulated by numerous afferent connections, including glutamatergic and GABAergic projections, as well as local GABAergic neurons (Adell and Artigas, 2004). In addition, the firing rates of DAergic neurons of the VTA are regulated by autoreceptors that inhibit the firing of these neurons (Grace, 1987).

There are predominantly 5 subtypes of DA receptors. There is structural homology between the D1 and D5 receptors, and both of these receptors are generally linked to Gαs, and stimulation of these receptors increases cAMP formation. The other 3 DA receptor subtypes (D2, D3, and D4) are structurally homologous and have been linked to Gαi and to reduction in cAMP formation (Neve et al., 2004). The autoreceptor on DAergic VTA neurons is of the D2 subtype, and activation of this receptor in mesencephalic DA neurons reduces the firing frequency of these neurons through a direct interaction of its G-protein with somatic membrane potassium channels (Kim et al., 1995; Lacey et al., 1988).

Ethanol (EtOH) has numerous specific actions on DA VTA neurons. In the VTA, acute EtOH increases h-current (Brodie and Appel, 1998), reduces M-current (Koyama et al., 2007), and increases barium-sensitive potassium current (McDaid et al., 2008). In addition, EtOH increases glutamatergic (Deng et al., 2009) and increases GABAergic postsynaptic potentials (Theile et al., 2008). Some actions of EtOH may directly cause the phenotypic response to EtOH (e.g., increased firing; Brodie et al., 1990; Gessa et al., 1985) and other effects may not directly play a role (McDaid et al., 2008), but may be involved in modulating those direct effects; the direct and modulatory effects may play a role in adaptation to EtOH subsequent to chronic (intermittent, repeated, or continuous) administration (Okamoto et al., 2006). A careful assessment of the acute effects of EtOH on these important neurons is the first step in understanding how EtOH actions in the VTA are related to the development of alcoholism.

We recently reported a time-dependent and concentration-dependent desensitization of DA D2 receptors requiring concurrent activation of D1-like and D2-like receptors which we called DA inhibition reversal (DIR; Nimitvilai and Brodie, 2010), and this desensitization is mediated by phospholipase C and the protein kinase C (PKC) pathway (Nimitvilai et al., 2012). The present report describes experiments to determine whether EtOH interacts with DIR.

## Materials and Methods

### Animals

Fischer 344 (F344; 90 to 150 gram) rats used in these studies were obtained from Harlan Sprague-Dawley (Indianapolis, IN). All rats were treated in strict accordance with the NIH Guide for the Care and Use of Laboratory Animals, and all experimental methods were approved by the Animal Care Committee of the University of Illinois at Chicago.

### Preparation of Brain Slices

Brain slices containing the VTA were prepared from the subject animals as previously described (Brodie et al., 1999a). Briefly, following rapid removal of the brain, the tissue was blocked coronally to contain the VTA and substantia nigra. The tissue block was mounted in the vibratome and submerged in chilled cutting solution. Coronal sections (400 μm thick) were cut, and the slice was placed onto a mesh platform in the recording chamber. The slice was totally submerged in artificial cerebrospinal fluid (aCSF) maintained at a flow rate of 2 ml/min at a temperature of 35°C. The composition of the aCSF in these experiments was (in mM): NaCl 126, KCl 2.5, NaH_2_PO_4_ 1.24, CaCl_2_ 2.4, MgSO_4_ 1.3, NaHCO_3_ 26, and glucose 11. The composition of the cutting solution was (in mM): KCl 2.5, CaCl_2_ 2.4, MgSO_4_ 1.3, NaHCO_3_ 26, glucose 11, and sucrose 220. Both solutions were saturated with 95% O_2_/5% CO_2_ (pH = 7.4). Equilibration time of at least 1 hour was allowed after placement of tissue in the chamber before electrodes were placed in the tissue.

### Cell Identification

The VTA was clearly visible in the fresh tissue as a gray area medial to the darker substantia nigra and separated from the nigra by white matter. Recording electrodes were placed in the VTA under visual control. Putative dopaminergic (pDAergic) neurons have been shown to have distinctive electrophysiological characteristics (Grace and Bunney, 1984; Lacey et al., 1989). Only those neurons that were anatomically located within the VTA and that conformed to the criteria for pDAergic neurons established in the literature and in this laboratory (Lacey et al., 1989; Mueller and Brodie, 1989) were studied. These criteria include broad action potentials (2.5 ms or greater, measured as the width of the bi- or triphasic waveform at the baseline), slow spontaneous firing rate (0.5 to 5 Hz), and a regular interspike interval. Cells were not tested with opiate agonists as has been done by other groups to further characterize and categorize VTA neurons (Margolis et al., 2006). It should be noted that some neurons with the characteristics we used to identify DA VTA neurons may not, in fact, be DA-containing (Margolis et al., 2006), and therefore, the DA VTA neurons are referred to as putative DA (pDA) VTA neurons. A recent study indicates that a high percentage of neurons in the VTA with electrophysiological characteristics of DA cells are, in fact, DAergic (Chieng et al., 2011).

### Drug Administration

Drugs were added to the aCSF by means of a calibrated infusion pump from stock solutions 100 to 1,000 times the desired final concentrations. The addition of drug solutions to the aCSF was performed in such a way as to permit the drug solution to mix completely with aCSF before this mixture reached the recording chamber. Final concentrations were calculated from aCSF flow rate, pump infusion rate, and concentration of drug stock solution. The small volume chamber (about 300 μl) used in these studies permitted the rapid application and washout of drug solutions. Typically, drugs reach equilibrium in the tissue after 2 to 3 minutes of application.

In some experiments, drugs were added to the microelectrode filling solution (0.9% NaCl) at a concentration 10 times greater than that which would have been used in the extracellular medium. To allow time for the drug to diffuse from the pipette to the cell, the effects of bath-applied drugs were tested no less than 20 minutes after initiating the recording. When both drug application methods were tested, this pipette-application method has produced results comparable to the bath-application method (data not shown), with the advantage of more localized drug application and reduced expense. Such local delivery of drugs through recording pipettes has been used in the past by our laboratory and others (Nimitvilai et al., 2012; Pesavento et al., 2000; Sokolov and Kleschevnikov, 1995).

DA hydrochloride, EtOH, quinpirole, and most of the salts used to prepare the extracellular media were purchased from Sigma (St. Louis, MO). Phorbol 12-myristate 13-acetate (PMA) and Gö6976 were purchased from Tocris (Ellisville, MO).

### Extracellular Recording

Extracellular recording was chosen for these studies as this method permits the recordings to be stable and of long duration (routinely >1.5 hours) and allows us to assess the effects of extended exposure (>30 minutes) to drugs. The limitation of only measuring spontaneous action potential frequency (rather than membrane potential or other electrophysiological parameters) is counterbalanced by the advantage of being able to determine the time course of drug actions and interactions. Extracellular recording electrodes were made from 1.5-mm-diameter glass tubing with filament and were filled with 0.9% NaCl. Tip resistance of the microelectrodes ranged from 2 to 4 MΩ. Offline analysis was used to calculate, display, and store the frequency of firing at 1-minute and 5-second intervals. Firing rate was determined before and during drug application. Firing rate was calculated over 1-minute intervals prior to administration of drugs and during the drug effect; peak drug-induced changes in firing rate were expressed as the percent change from the control firing rate according to the formula ((FRD − FRC)/FRC) × 100, where FRD is the firing rate during the peak drug effect, and FRC is the control firing rate. The change in firing rate thus is expressed as a percentage of the initial firing rate, which controls for small changes in firing rate which may occur over time. For example, the effect of DA over time is compared with the pre-DA baseline; in cases in which the concentration of DA was increased to achieve >50% inhibition, the firing rates are compared with the baseline before any DA was added to the superfusate. This formula was used to calculate both excitatory and inhibitory drug effects. Peak excitation was defined as the peak increase in firing rate produced by the drug (e.g., DA) greater than the predrug baseline. Inhibition was defined as the lowest firing rate below the predrug baseline. Inhibition reversal was observed as a statistically significant reduction in the inhibition.

### Data Collection

For comparison of the time course of effects on firing rate, the data were normalized and averaged. Firing rates over 1-minute intervals were calculated and normalized to the 1-minute interval immediately prior to the DA administration. These normalized data were averaged by synchronizing the data to the DA administration period, and graphs of the averaged data were plotted.

### Statistical Analysis

**Fig. 1:**
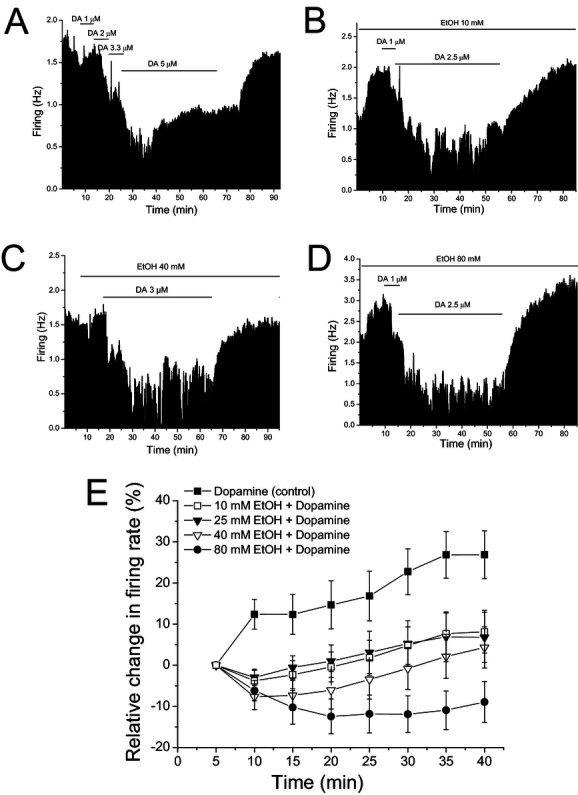
Ethanol (EtOH) prevents dopamine (DA) inhibition reversal (**A–D**). Mean ratemeter graphs of the effects of a 40-minute DA application under different conditions on 4 different pDAergic ventral tegmental area neurons. Vertical bars indicate the firing rate over 5-second intervals. Horizontal bars indicate the duration of drug application (concentrations indicated above bar). (**A**) DA concentration was increased in a stepwise fashion until >50% inhibition was achieved, and then, that concentration was applied for a total of 40 minutes. Over time, the DA-induced inhibition decreased. (**B**) In the presence of 10 mM EtOH, there was no obvious reduction in the effect of DA applied for 40 minutes until the last 10 minutes. (**C**) In the presence of 40 mM EtOH, no obvious decrease in the effect of DA over the 40-minute application period was observed. (**D**) In the presence of 80 mM EtOH, there was a slight increase in the DA-induced inhibition over the 40-minute time period of DA application. (**E**) Pooled time course graph for the cells tested for DIR in the absence of (▪, control, [DA] = 5.5 ± 1.0 μM, *n* = 25) or in the presence of EtOH (10 (□, [DA] = 5.8 ± 0.6 μM, *n* = 28), 25 (▼, [DA] = 5.4 ± 0.8 μM, *n* = 24), 40 (∇, [DA] = 5.0 ± 0.7 μM, *n* = 20), and 80 (•, [DA] = 5.0 ± 0.9 μM, *n* = 15) mM). Time indicated on the abscissa reflects time relative to the initiation of DA administration.

**Fig. 2:**
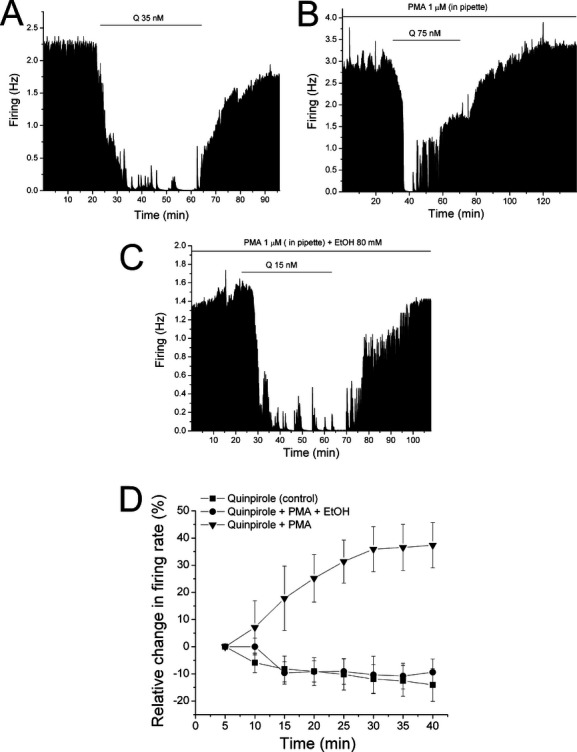
Ethanol (EtOH) prevents reversal of quinpirole (Q) inhibition induced by phorbol 12-myristate 13-acetate (PMA). (**A–C)** Mean ratemeter graphs of the effects of a 40-minute Q application under different conditions on 3 different pDAergic ventral tegmental area neurons. Vertical bars indicate the firing rate over 5-second intervals. Horizontal bars indicate the duration of drug application (concentrations indicated above bar). (**A**) Application of Q (35 nM) produced >50% inhibition and was applied for a total of 40 minutes. No reduction in the inhibitory effect of Q was observed over the 40-minute time period. (**B**) Application of Q (75 nM), in a recording in which PMA (1 μM) was in the pipette, produced >50% inhibition, and was applied for a total of 40 minutes. Substantial reduction in Q-induced inhibition was observed during the 40-minute application period. (**C**) Application of Q (15 nM), in a recording in which PMA (1 μM) was in the pipette and 80 mM EtOH was in the superfusate, produced >50% inhibition and was applied for a total of 40 minutes. No obvious reduction in Q-induced inhibition was observed during the 40-minute application period. (**D**) Pooled time course graph for the cells tested with Q with normal saline in the pipette (▪, [Q] = 35.8 ± 3.0 nM, *n* = 6), with 1 μM PMA in the pipette (▼, [Q] = 50.0 ± 15.9 nM, *n* = 5), and in the presence of 80 mM EtOH with 1 μM PMA in the pipette (•, [Q] = 38.3 ± 6.7 nM, *n* = 6). Time indicated on the abscissa reflects time relative to the initiation of Q administration.

**Fig. 3:**
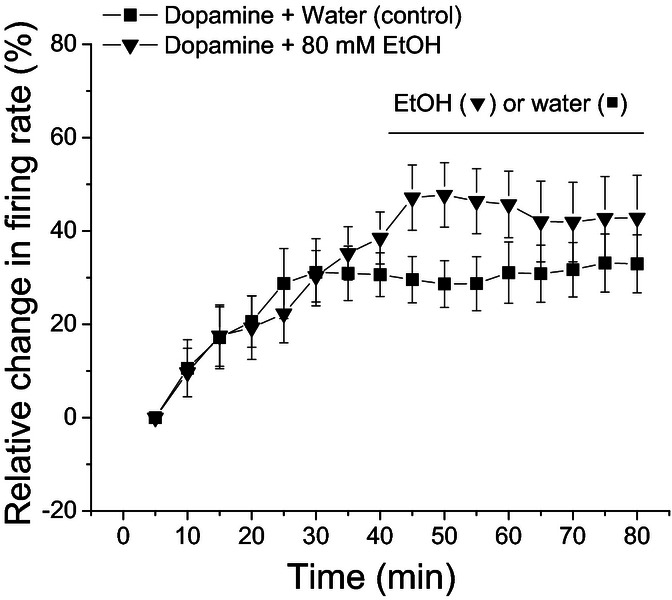
Ethanol (EtOH) fails to antagonize dopamine inhibition reversal (DIR) once it has been induced. Pooled results from recordings of pDAergic ventral tegmental area (VTA) neurons tested with dopamine (DA) over 80 minutes. Time indicated on the abscissa reflects time relative to the initiation of DA administration. For each neuron, a concentration of DA that produced >50% inhibition was applied for 80 minutes. After DIR was induced within the first 40 minutes, either 80 mM EtOH (▼; [DA] = 6.3 ± 0.8 μM, *n* = 12) or water (▪, [DA] = 5.3 ± 1.3 μM, *n* = 7) was added to the superfusate, and the recording was continued for 40 more minutes. No significant difference (1-way repeated measures ANOVA, *F*(16, 287) = 008, *p* > 0.005, for comparisons between EtOH and water conditions) in the effect of DA was observed from 40 to 80 minutes, indicating that once it is achieved, DIR is not antagonized by EtOH.

**Fig. 4:**
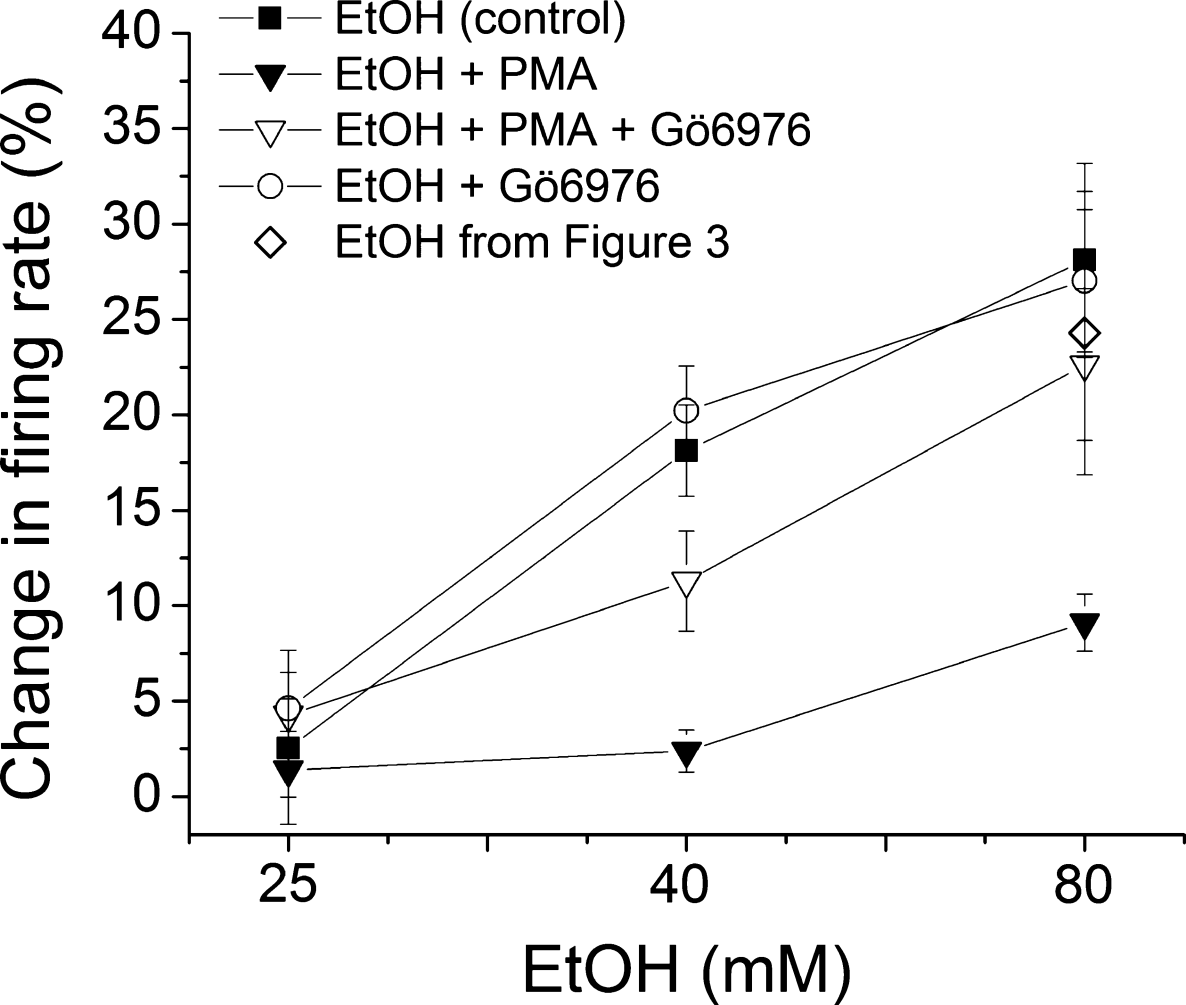
Phorbol 12-myristate 13-acetate (PMA) but not Gö6976 reduces ethanol (EtOH) excitation. Pooled results from recordings of pDAergic ventral tegmental area (VTA) neurons tested with EtOH (25, 40, and 80 mM). For each neuron, each concentration of EtOH was applied in the extracellular medium for 6 minutes and washed out for at least 10 minutes before the next concentration of EtOH was tested. Recordings were made with normal saline alone or normal saline containing either PMA (1 μM), Gö6976 (10 μM), or both drugs, in the recording pipette. In recordings with electrodes containing saline alone (▪), or saline with PMA plus Gö6976 (∇), or saline with Gö6976 alone (○), the excitatory effects of EtOH were not statistically different; in recordings with PMA (▼) alone in the pipette, the effect of EtOH was attenuated compared with all the other groups (2-way ANOVA, *F*(3, 109) = 13.6, *p* < 0.05; *p* < 0.05 for difference between PMA and all other conditions, *p* > 0.05 for comparisons among all other conditions). Also plotted are data from Fig. 3 of the excitatory effect of 80 mM EtOH after the 40-minute administration of dopamine (◊).

Averaged numerical values were expressed as the mean ± the standard error of the mean (SEM). The differences among firing rates during the long drug administration intervals in these studies were assessed with 1-way repeated measures analysis of variance (ANOVA) (Figs 1 and 2) or 2-way ANOVA (Figs 3 and 4), followed by Tukey's post hoc comparisons (Kenakin, 1987). Pooled data in Figs 3 are expressed as relative change in firing rate; this permits normalization of the data to the inhibitory effect of DA at the first time point (5 minutes) and reflects the statistical analysis, which compared the effects of DA at each time point over time. Time-dependent effects of DA for populations of neurons (Figs 1*E*, 2*D*, and 3) are expressed as time relative to the initiation of DA administration; ratemeter recordings illustrate time during the experiment, with drug onset illustrated by horizontal bars. Pooled data in Fig. 4 were expressed as change in firing rate as a percentage of pre-EtOH baseline. Statistical analyses were performed with Origin 8.5 (Originlab Corporation, Northampton, MA).

## Results

### VTA Neuron Characteristics

A total of 188 VTA neurons were examined. Their firing rate in normal extracellular medium ranged from 0.48 to 4.17 Hz, with a mean of 1.74 ± 0.05 Hz. All neurons had regular firing rates and were inhibited by DA agonists. Sensitivity to DA (0.5 to 5 μM) was initially assessed by administering the agonist for 5 minutes and then washing it out until the firing rate recovered to at least 70% of the baseline firing rate; quinpirole (25 to 150 nM) was administered for 5 minutes, and the concentration was increased if inhibition >50% was not achieved. The concentrations of agonist were adjusted for each neuron so that inhibition exceeded 50%, as inhibition that was <50% was not reliably reversed (Nimitvilai and Brodie, 2010). This method of adjusting the concentration of DAergic agonist controlled for differences in sensitivity between neurons, but also sometimes resulted in the mean concentrations of DA or quinpirole slightly differing between groups. Overall, for pDAergic VTA neurons from adult rats, the concentration of DA used was 5.48 ± 0.32 μM (*n* = 131), which produced a mean change in firing rate of −74.5 ± 1.3%; the concentration of quinpirole used was 43.8 ± 7.5 nM (*n* = 17), which produced a mean change in firing rate of −65.8 ± 3.1%. For pDA VTA neurons to which DA was administered for 40 minutes in the absence of other agents, reversal of DA inhibition was observed in 39 of 47 cells (83.0%); this is similar to the percentage of pDA VTA neurons that exhibited DIR in our initial report (Nimitvilai and Brodie, 2010). There were no significant differences in the concentration of DAergic agonists, 1-way ANOVA, *F*(4, 107) = 0.18, *p* > 0.05, or in the percent inhibition, 1-way ANOVA, *F*(4, 107) = 1.42, *p* > 0.05, among the groups. Cells that did not return to at least 70% of their pre-DA firing rate during this washout were not used. One benefit of the extracellular recording method used in these studies is that long duration recordings can be made reliably; the average recording duration was 91.5 ± 0.7 minutes, with a range of 80 to 120 minutes.

EtOH was administered 10 minutes prior to application of DA agonists. The mean excitatory effect of EtOH was concentration dependent, as reported previously (Brodie et al., 1990). The mean excitation (%) by EtOH in the experiments with DA was 4.4 ± 2.8 for 10 mM, 6.9 ± 1.4 for 25 mM, 15.4 ± 2.9 for 40 mM, and 18.3 ± 2.9 for 80 mM. These levels of excitation were similar to those previously reported by us for the effects of these EtOH concentrations (Brodie et al., 1990).

### EtOH Decreases DIR

In the presence of EtOH, DIR is reduced. Figure 1 illustrates the effect of EtOH on inhibition by DA over a 40-minute time period in the absence and presence of various concentrations of EtOH. As in our previous study, in the absence of EtOH (Control) (Fig. 1*A,E*), the inhibitory effect of DA decreases over time, so that firing rates from 10 to 40 minutes are significantly different from the firing rate at 5 minutes after initiation of the DA administration ([DA] = 5.5 ± 1.0 μM, *n* = 25), 1-way repeated measures ANOVA, *F*(1, 24) = 197.2, *p* < 0.05, for differences between 5 minutes and all other time points. In the presence of a relatively low concentration of EtOH (10 mM, Fig. 1*B,E*), only the 40-minute time point was significantly different from the 5-minute time point administration ([DA] = 5.8 ± 0.6 μM, *n* = 28), 1-way repeated measures ANOVA, *F*(1, 27) = 329.8, *p* < 0.05, for differences between 5 and 40 minute time points. At 25 mM EtOH (Fig. 1*E*), there was no significant difference in the effect of DA among the time points over the 40-minute administration ([DA] = 5.4 ± 0.8 μM, *n* = 24), 1-way repeated measures ANOVA, *F*(1, 23) = 305.9, *p* > 0.05, for differences between 5 minutes and all other time points. At 40 mM EtOH (Fig. 1*C,E*), there was no significant difference between the 5-minute time point and any of the other time points, although there was a significant difference from the 40-minute time point for each of the 10-, 15-, and 20-minute time points ([DA]A = 5.0 ± 0.7 μM, *n* = 20), 1-way repeated measures ANOVA, *F*(1, 19) = 305.9, *p* < 0.05, for differences between 10 to 20-minute and 40-minute time points). At 80 mM EtOH (Fig. 1*D,E*), there was a significant difference between the 5-minute time point and time points from 15 to 40 minutes ([DA] = 5.0 ± 0.9 μM, *n* = 15), 1-way repeated measures ANOVA, *F*(1, 14) = 305.9, *p* < 0.05, for differences between 5-minute time point and 15 to 40-minute time points, but as can be seen from Fig. 1*E*, the inhibitory effect of DA actually increased over time, so there was greater inhibition from 15 to 40 minutes compared with the 5-minute time point.

### EtOH Attenuates the Reversal of Quinpirole Inhibition by PKC Activator

DIR is dependent on activation of PKC and that activation of PKC with a phorbol ester can cause reversal of inhibition induced by the selective D2 agonist quinpirole without the addition of a D1/D5 agonist (Nimitvilai et al., 2012). In Fig. 2, we illustrate that effect. In Fig. 2*A*, a typical pDAergic VTA neuron is inhibited by 35 nM quinpirole and that inhibition did not decrease over the 40-minute time of application of quinpirole; the 35 and 40 minutes showed significantly greater inhibition than at the 5-minute time point (Fig. 2*A,D*; [quinpirole] = 35.8 ± 3.0 nM, *n* = 6; 1-way repeated measures ANOVA, *F*(1, 5) = 17.5, *p* < 0.05, for differences between 5 minute and 35 to 40 minute time points); this increase in quinpirole inhibition with time has been reported (Nimitvilai and Brodie, 2010). Figure 2*B* illustrates an experiment in which a typical pDAergic VTA neuron is exposed similarly to quinpirole, but in this case, a phorbol ester, PMA (1 μM), is included in the pipette. In this case, the inhibitory effect of quinpirole is reduced over time; there was a significant difference between the 5-minute time point and all of the subsequent time points (Fig. 2*B,D*; [quinpirole] = 50.0 ± 15.8 nM, *n* = 5; 1-way repeated measures ANOVA, *F*(1, 4) = 3.30, *p* < 0.05, for differences between 5 minutes and all subsequent time points). In contrast, in the presence of 80 mM EtOH, even with PMA in the electrode, no reversal of quinpirole inhibition is observed over time, with a slightly greater inhibition at 30 minutes than at 5 minutes (Fig. 2*C,D*; [quinpirole] = 38.3 ± 6.7 nM, *n* = 6; 1-way repeated measures ANOVA, *F*(1, 5) = 1.77, *p* < 0.05, for a difference between 5- and 30-minute time points). The pooled data from these experiments are illustrated in Fig. 2*D*. A significant reduction in inhibition was observed in experiments in which quinpirole was tested in recordings with PMA in the pipette (▼), but not with control pipette solution (▪) or with PMA in the pipette and 80 mM EtOH in the bath (•).

### EtOH does not Antagonize DIR once it has Developed

Induction of DIR by DA requires stimulation of both D2 and D1/D5 DA receptors (Nimitvilai and Brodie, 2010). This reduction in sensitivity to D2 DA receptor-mediated inhibition lasts for up to 90 minutes after DA washout and cannot be antagonized by a D1/D5 antagonist once it develops (Nimitvilai and Brodie, 2010). We tested whether application of EtOH after DIR has developed can antagonize DIR. DA alone was applied for 40 minutes, and then, DA administration was continued in the presence of either EtOH or water for an additional 40 minutes. As shown in Fig. 3, there was significant reversal of the inhibitory effect of DA in the first 40 minutes, but there was no change in the sensitivity produced by DA in the second 40-minute time period in the presence of EtOH ([DA] = 6.3 ± 0.8 μM, *n* = 12) compared with the effect of DA in the presence of water control ([DA] = 5.3 ± 1.3 μM, *n* = 7), 2-way ANOVA, *F*(16, 287) = 0.08, *p* > 0.05, for a difference between EtOH and water control; *p* < 0.05 for effect of time on DA inhibition), indicating that EtOH did not antagonize DIR once it developed.

### EtOH Excitation is Reduced by Activation of PKC

As the primary effect of EtOH is to increase the firing of pDA VTA neurons (Brodie et al., 1990), it is possible that extended activation of PKC by DAergic receptors (Nimitvilai et al., 2012) could alter the direct effect of EtOH on firing rate. We assessed whether EtOH excitation was altered by activation of PKC with PMA. Recordings were made with normal saline alone or normal saline containing either 1 μM PMA, Gö6976 (10 μM), or both drugs, in the recording pipette. The results of these experiments are shown in Fig. 4. EtOH alone (25 to 80 mM) produced a concentration-dependent increase in the spontaneous firing frequency (▪). When PMA was included in the micropipette (▼), the excitatory effect of EtOH was significantly reduced, 2-way ANOVA, *F*(3, 109) = 13.6; *p* < 0.05 for difference between PMA and all other conditions, *p* > 0.05 for comparisons among all other conditions. In experiments in which Gö6976 and PMA (∇) were included in the pipette, there was no significant difference from the effect of EtOH with saline in the pipette. Likewise, there was no significant change in the excitatory effect of EtOH when Gö6976 alone (○) was included in the pipette compared with control. In addition, in the experiments summarized in Fig. 3, the effect of EtOH after 40 minutes DA was assessed at 6 minutes, and that value (◊ at 23.3 ± 7.4%) is graphed in Fig. 4 as well. These results indicate that activation of PKC by PMA can reduce EtOH excitation, but the processes involved in DIR (including activation of PKC) appear to be insufficient to alter EtOH-induced excitation.

## Discussion

The results presented here indicate that a low concentration of EtOH can interfere with desensitization of pDAergic VTA neurons. Human blood alcohol concentrations at legal intoxication in the United States (0.08 mg%) is equivalent to about 17 mM, and rats appear to be less sensitive to the effects of EtOH than humans (Haggard et al., 1940; Majchrowicz and Hunt, 1976), so the concentrations at which EtOH reduces DIR are pharmacologically relevant. We have previously reported a phenomenon of DIR with extended periods of exposure to moderate concentrations of DA, which differs from homologous and heterologous desensitization as it requires the concurrent stimulation of D1/D5 and D2 DA receptors (Nimitvilai and Brodie, 2010). We have begun characterizing this phenomenon and have found that it is dependent on PKC, but not on cAMP or protein kinase A, is sensitive to external calcium concentration, and is dependent on intracellular calcium release (Nimitvilai et al., 2012). Pure DA D2 agonists like quinpirole do not exhibit DIR, but reversal of quinpirole inhibition can be observed in the presence of D1/D5 agonists (Nimitvilai and Brodie, 2010) or activators of PKC like PMA (Nimitvilai et al., 2012). Our observation that EtOH blocks the reversal of quinpirole inhibition in the presence of PMA indicates that EtOH interferes with DIR at a step at or beyond the activation of PKC.

The action of EtOH on PKC is a possible mechanism by which it blocks DIR. There are numerous reports examining the action of EtOH on PKC (for review, see Newton and Messing, 2006; Stubbs and Slater, 1999). Acute EtOH can inhibit activation of PKC by interfering with translocation to the plasma membrane (Slater et al., 1993; Steiner et al., 1997). The effect of EtOH on PKC differs by subtype of PKC, as PKCα (Reneau et al., 2011; Slater et al., 1997), PKCγ (Harris et al., 1995; Rex et al., 2008), and PKCδ (Rex et al., 2008) have been reported to be inhibited by EtOH, but other isoforms are resistant to EtOH inhibition, like PKCβ1 and PKCε, for example (Rex et al., 2008). Other laboratories have shown that EtOH can enhance the activity of some PKC isoforms, such as PKCε (Messing et al., 1991; Satoh et al., 2006). The effect of EtOH on PKC isoforms can be tissue specific, as EtOH decreases the activity of PKCδ in HEK293 cells (Rex et al., 2008) but increased PKCδ activity in neural PC12 cells (Messing et al., 1991). One study found that the function of highly purified PKC was not reduced by EtOH (Machu et al., 1991). Additional studies will be necessary to ascertain whether EtOH acts on PKC to disrupt DIR in pDA VTA neurons and whether other actions of EtOH contribute to this disruption.

Once DIR is activated, it is not reversed (i.e., sensitivity to DA-induced inhibition is not returned to initial levels) by application of D1/D5 antagonists (Nimitvilai and Brodie, 2010). This indicates a long-lasting change in processes that regulate the sensitivity of the response to D2 agonists, such as a sustained change in the phosphorylation state of the D2 receptor or in the surface expression of that receptor. Alternatively, it could be due to other intracellular processes (like PKC activation state) that are initiated by concurrent D1/D5 and D2 stimulation and that persist after those processes have been triggered. As EtOH application following DIR induction did not restore sensitivity to DA-induced inhibition (Fig. 3), the mechanisms by which EtOH disrupts DIR does not influence expression of DIR once it is induced. As additional studies elucidate the molecular pathways involved in the mechanism of DIR, the points at which EtOH interferes with those pathways may become clearer.

As the reduction in desensitization to DA-induced inhibition can be blocked by low-to-moderate concentrations of EtOH, acute administration of EtOH may cause a functional enhancement of inhibition by endogenous DA in the presence of EtOH. The net effect of this action of EtOH would be to decrease the excitability of pDAergic VTA neurons, making them less sensitive to excitatory neurotransmitters or other factors. The primary effect of EtOH on DA VTA neurons is excitation, whether it is measured in vivo (Gessa et al., 1985) or in vitro (Brodie et al., 1990). Excitation is due to a direct action on the DA neurons (Brodie et al., 1999b) and may be the result of EtOH blockade of some potassium channels, including M-current (Koyama et al., 2007). The antagonism by acute EtOH of DIR would act to undermine that direct excitation. EtOH itself increases the concentration of DA in the VTA over time (Kohl et al., 1998), and the inhibitory effect of that DA would be maintained (would not exhibit desensitization) in the presence of EtOH, as we show here. Like the effect of EtOH on barium-sensitive potassium channels (McDaid et al., 2008), this would tend to decrease the excitatory effects of acutely administered EtOH in naïve subjects.

The acute and chronic actions of EtOH on regulation of autoreceptor sensitivity of pDAergic VTA neurons may be important for determining initial sensitivity as well as chronic response of these neurons to EtOH. As noted previously, this action of EtOH would tend to decrease the acute excitatory response to EtOH. Initial low sensitivity to the effects of EtOH has been correlated with a higher risk of the development of alcoholism (Schuckit, 1994). The reduction of DIR induced by acute EtOH would reduce the activity of DAergic neurons of the VTA and might have a variety of effects on motor activity. It may be that reduced sensitivity to EtOH blockade of DIR may be related to increased risk of alcoholism. Extensive additional studies would be needed to determine whether the variant of the D2 receptor (Blum et al., 1991; Curtis et al., 1999) or associated gene products (Dick et al., 2007) that are correlated with risk to alcoholism differ in sensitivity to desensitization or to EtOH action on the processes related to desensitization, respectively. As genetic variants in D2 receptors are linked to other psychiatric diseases (Noble, 2003), these variants may also differ in their sensitivity to DIR.

As DIR appears to be an expression of desensitization, our results show a very potent effect of EtOH on that desensitization. During withdrawal from chronic EtOH exposure, the activity of DA VTA neurons has been reported to be reduced (Diana et al., 1992); others have reported an increased number of silent DA neurons in the VTA (Shen and Chiodo, 1993). Desensitization of D2 receptors in the VTA has been reported to be reduced in mice after 7 days of repeated intraperitoneal injection of EtOH, which resulted in greater autoinhibition and reduced DA tone (Perra et al., 2011). In contrast, an increase in DA release in nucleus accumbens and caudate/putamen was observed in rats after exposure to EtOH vapor for 5 to 10 days (Budygin et al., 2007); in this study, there was a lack of effect of quinpirole on DA efflux in the nucleus accumbens, suggesting a lack of change in autoreceptor regulation of DA release. As we show here that acute EtOH potently reduces desensitization of D2 autoreceptors, chronic or repeated EtOH may induce adaptive changes in regulation of DA neuronal activity to compensate for the lack of desensitization of D2 receptors in the persistent presence of EtOH. As the studies noted previously use different species as well as different methods of EtOH exposure, additional studies will be necessary to examine the mechanisms of adaptation to persistent block of DIR by EtOH and to determine whether the mode of EtOH administration influences how the physiology of DA VTA neurons adapts to the persistent presence of EtOH.

In VTA neurons from mice, autoreceptor inhibition is attenuated after chronic EtOH (Perra et al., 2011). In that study, the desensitization appeared to be linked to calmodulin kinase II and not to PKC; desensitization to DA in rat pDA VTA neurons is clearly dependent on PKC (Nimitvilai et al., 2012). We show here that the processes that result in the production of DIR by DA do not result in the reduction of EtOH excitation, but activation of PKC by PMA did in fact result in a reduction of sensitivity to EtOH excitation. This paradox may be due to a more selective activation of PKC by DA or by a more potent activation of PKC by PMA than by DA. The calcium dependence of DIR, and blockade of DIR by the conventional PKC antagonist Gö6976, suggests the involvement of a conventional PKC (Nimitvilai et al., 2012), whereas acute or chronic EtOH can affect some isoforms of novel PKC, including PKCε (Jiang and Ye, 2003) and PKCδ (Gerstin et al., 1998) (for review, please see Newton and Messing, 2006). Many additional studies would be necessary to identify the cellular elements altered by PKC that result in the decrease in EtOH excitation in DA VTA neurons. The role of EtOH to block DIR may reveal important actions of acute EtOH to disrupt information processing in the VTA. Examination of this action of EtOH on pDAergic VTA neurons may permit a better understanding of the array of actions of EtOH on these neurons, especially when the acute actions of EtOH are compared with the effects of EtOH after chronic alcohol administration.
